# Impact of comorbidities on patient-reported outcomes in psoriatic arthritis: a single centre cohort study

**DOI:** 10.1007/s00296-024-05632-2

**Published:** 2024-06-25

**Authors:** Grzegorz Biedroń, Mateusz Wilk, Jarosław Nowakowski, Piotr Kuszmiersz, Zofia Guła, Magdalena Strach, Alen Brkic, Glenn Haugeberg, Mariusz Korkosz

**Affiliations:** 1 Department of Rheumatology and Immunology, Jagiellonian University Medical College, Jakubowskiego 2, Kraków, Poland; 2 Department of Rheumatology, Clinical Immunology and Internal Medicine, University Hospital in Cracow, Kraków, Poland; 3 Division of Rheumatology, Department of Internal Medicine, Sørlandet Hospital, Kristiansand, Norway; 4 Department of Neuromedicine and Movement Science, Faculty of Medicine and Health Sciences, NTNU, Norwegian University of Science and Technology, Trondheim, Norway

**Keywords:** Psoriatic arthritis, Comorbidity, Multimorbidity, Patient reported Outcome measure, Quality of life

## Abstract

**Background:**

Comorbidities are frequent in psoriatic arthritis (PsA) and may contribute to worse health-related outcomes. Patient-reported outcomes (PROs) are used to evaluate the burden of the assessed disease. The aim of this study is to evaluate the impact of comorbidities on selected PROs in PsA.

**Methods:**

Adult patients, diagnosed with PsA, based on CASPAR criteria, were included in this cross-sectional, observational study. Collected data encompassed the comorbidities and PROs (Health Assessment Questionnaire [HAQ], Multi-Dimensional Health Assessment Questionnaire [MDHAQ], 36-Item Short Form Health Survey [SF-36]). Standard statistic methods were performed for data assessment.

**Results:**

There were 267 participants included in the study (54.7% females). The most prevalent comorbidities were cardiovascular diseases (CVD) (29.2 %). Multimorbidity was observed in 50.2% cases and was associated with poorer results of SF-36 questionnaire, regarding bodily pain (34.7 [30.1, 39.3] vs. 47.5 [43.1, 52.0]; *p*<0.01), physical functioning (52.1 [47.3, 56.9] vs. 63.1 [58.9, 67.4]; *p*<0.01) and role physical (28.5 [21.2, 35.9] vs. 42.8 [35.2, 50.4]; *p*<0.01). CVD were associated with poorer MDHAQFn score (β=0.17, *p*<0.01), while mental disorders negatively influenced mental health (β= -0.35, *p*<0.01), vitality (β= -0.22, *p*<0.01), general health (β= -0.19, *p*<0.01), social functioning (β= -0.15, *p*=0.04) and role emotional (β= -0.30, *p*<0.01) dimensions of SF-36.

**Conclusions:**

Multimorbidity exerts significant impact on physical aspects of quality of life (QoL) in PsA. CVD and mental disorders adversely influence functional capacity as well as mental and social dimensions of QoL, respectively. The impact of comorbidities should be taken into account by clinicians and researchers assessing PROs.

**Supplementary Information:**

The online version contains supplementary material available at 10.1007/s00296-024-05632-2.

## Introduction

### Psoriatic arthritis

Psoriatic arthritis (PsA) is a chronic, inflammatory disease, which belongs to the group of spondyloarthritis (SpA). It is characterized by the presence of asymmetric peripheral arthritis, enthesitis, dactylitis, spine involvement and frequently associated with skin and nail psoriasis [1, 2]. The prevalence of this entity is estimated to be 113–133 per 100 000 subjects [3, 4]. Unrecognized and untreated PsA may lead to severe consequences, including erosions forming, joint deformation and, according to some reports, even higher mortality [5–7]. State-of-the-art management of PsA, as well as of the other inflammatory arthritides, involves a treat-to-target strategy [8, 9], which demands precise assessment of disease activity. Such evaluation includes not only objective factors (i.e., number of swollen joints, radiologic images, inflammatory parameters like C-reactive protein (CRP)) but also subjective ones based on the patient’s opinion. Moreover, the disease burden goes beyond the straight consequences of inflammation and encompasses the impact on quality of life (QoL), functionality, mental health, and social functioning [6, 8, 10].

### Comorbidities

Comorbidities are common in PsA, similarly to the other rheumatic diseases. Comorbid diseases may appear as a result of shared pathogenetic factors or applied treatment for one of the disorders but may be also not specifically associated. Among the most prevalent comorbidities are hypertension and other cardiovascular diseases (CVD), obesity, metabolic/endocrine disorders such as diabetes mellitus or thyroid disease, depression, and mental health disorders [11–15]. . Their presence may interfere with the assessment of PsA activity by influencing the patient’s symptoms and signs. Some comorbidities may also contribute to worse outcomes and negatively impact the administered treatment [16, 17].

### Patient-reported outcomes

Patient-reported outcomes (PROs) are important tools used to assess the burden associated with rheumatic disease. They are commonly used in different inflammatory arthritides [18–20]. They include symptoms like pain, fatigue, and feeling of stiffness or validated questionnaires assessing different domains of health and QoL [21]. Several questionnaires may be used to assess PROs in rheumatic diseases. Health Assessment Questionnaire (HAQ) describes patient’s disability in the sections: dressing, arising, eating, walking, hygiene, reach, grip, and activities [22]. It was introduced for the assessment of the health status in PsA [23, 24]. Multi-Dimensional Health Assessment Questionnaire (MDHAQ) is extended version of HAQ, assessing functional - Multi-Dimensional Health Assessment Questionnaire Functional (MDHAQFn) - and psychological components - Multi-Dimensional Health Assessment Questionnaire Psychological (MDHAQPs) [25]. MDHAQ use was validated in PsA [26]. 36-Item Short Form Health Survey (SF-36) evaluates several domains of QoL such as mental health (MH), vitality (VT), bodily pain (BP), general health (GH), social functioning (SF), physical functioning (PF), role limitations due to physical problems (RP) and role limitations due to emotional problems (RE). It is commonly used in rheumatic diseases, including PsA [27, 28]. Nevertheless, PROs are based on patients’ subjective assessment, which renders them susceptible to confounders, including comorbidities, as many of the reported symptoms are not specific to inflammatory rheumatic diseases. The systematic review published by Canete et al. indicated a high prevalence and a high impact of comorbidities on PROs in PsA. However, it stated that more research on this matter is desirable [22].

## Aim of the study

Psoriatic arthritis is a complex disease and its optimal management entails taking into account PROs. To choose the appropriate interventions, it is important to evaluate, whether these subjective outcomes are associated mostly with the disease activity or the other factors. The main goal of this study is to assess the impact of comorbidities on patient-reported health status and QoL in PsA, based on data from a single Polish clinical centre. Consistently with the main goal, our study aimed at basic description of study population, analysis of the kind and prevalence of particular comorbidities, assessment of chosen PROs (SF36, HAQ, MDAQFn, MDHAQPs) and determining the association between comorbidities and PROs.

## Methods

### Study design

The study was performed as a cross-sectional, observational study based on data collected in the Department of Rheumatology and Immunology, Jagiellonian University, Medical College in Cracow.

### Study population

Adult (over 18 years old) patients treated in the rheumatology outpatient clinic of The University Hospital in Krakow, Poland, diagnosed with PsA on the basis of CASPAR criteria [30] were included. All consecutive cases from 5th March 2021 to 3rd November 2023, who signed written consent were involved in the study. There were no specific exclusion criteria. Two hundred and sixty – seven participants were recruited.

### Data collection and data variables

Data were collected using the GoTreatIt® Rheuma software and included in a structured database as a part of standard clinical care [31]. Comorbidity data were gathered methodically through a dual approach. Initially, patients provided information about their comorbid conditions through a structured questionnaire during their visit, followed by a thorough review of their electronic health records (EHR) to confirm these conditions. Subsequently, the treating physicians conducted detailed evaluations of the patient’s treatment histories to validate the presence of these comorbidities and their management. The data included demographic variables e.g., age, sex, body mass index (BMI, kg/m2), smoking status, physical activity level, disease duration, and clinical variables, e.g., C-reactive protein (CRP), presence of axial disease, number of tender and swollen joints, Disease Activity Index for Psoriatic Arthritis (DAPSA), Bath Ankylosing Spondylitis Disease Activity Index (BASDAI), and the presence and number of comorbidities. The data also included current treatment encompassing non-steroidal anti-inflammatory drugs (NSAIDs), glucocorticoids, conventional synthetic DMARDs (csDMARDs), and biological/targeted synthetic DMARDs (b/tsDMARDs). Lastly, the data also included patient’s reported variables, e.g. HAQ, MDHAQFn, MDHAQPs, and SF-36. A joint assessment was performed by doctors during routine visits, along with a collection of medical history, laboratory results, and treatment data. Demographic data and PROs were self-registered by the patients. Comorbidities were defined using standard definitions and categorized into distinct subgroups (e.g., cardiovascular, respiratory, gastrointestinal).

### Statistical analyses

Numerical variables are presented as mean with a 95% confidence interval (CI), while categorical variables are expressed as counts with corresponding percentages. The percentage of missing data is provided to indicate data density. Prior to analysis, the normality of distribution for examined variables was assessed using the Kolmogorov-Smirnov test alongside visual inspection of histograms. Since none of the tested variables exhibited a normal distribution, the Mann-Whitney U test was employed to compare the means. In order to investigate potential associations between comorbidities and Health-Related Quality of Life (HRQoL), multivariable linear regression with backward elimination was performed. A probability threshold (F-to-remove) greater than 0.20 was set for variable removal from the model [32]. HRQoL measures were designated as dependent variables while adjusting for covariates, including age, sex, BMI, disease duration, and physical activity. The adjusted R-squared (R²) values were calculated based on the remaining variables within the models. Additionally, the Variation Inflation Factor (VIF) was computed for all models to detect and address potential multicollinearity issues, ensuring all VIF values were below 4 in the final models [33]. Statistical analyses were conducted using IBM SPSS version 28, with significance set at a p-value of less than 0.05.

### Ethics approval and patient involvement

The survey’s research protocol was approved by the Institutional Review Board (IRB) of the Jagiellonian University Medical College (protocol N 118.6120.07.2023, June 15, 2023). All participants provided informed consent before completing the questionnaire, with the assurance that their responses would be used solely for research purposes.

## Results

There were 267 participants included in the study, 54.7% of whom were females. The mean age was 48.3 (46.7, 49.8) and the mean disease duration was 7.8 (6.8, 8.8) years. The DAPSA and BASDAI scores were 16.4 (14.4, 18.4) and 3.5 (3.0, 4.0), respectively, which indicates low to moderate disease activity. BASDAI score was calculated only for cases with axial disease. The basic characteristics of the group are presented in Table 1.

**Table 1:** Demographics, clinical data, and patient-reported questionnaire results from 267 participants with psoriatic arthritis

			Missing data (%)
**Demographics**	Age, years	48.3 (46.7, 49.8)	0%
	Women	146 (54.7%)	0%
	Body mass index, kg/m2	27.7 (27.0, 28.4%)	12%
	Cigarette smoker (ever)	105 (39.3%)	12.7%
	Physical exercise > 1 h/week	73 (27.3%)	2.6%
**Clinical information**	Axial disease	45 (16.9%)	0%
	Disease duration, years	7.8 (6.8, 8.8)	12.6%
	CRP	5.7 (4.6, 6.9)	4.5%
	66 swollen joint count	2.2 (1.7, 2.6)	10.1%
	68 tender joint count	6.2 (5.0, 7.4)	10.1%
	DAPSA	16.4 (14.4, 18.4)	18.4%
	BASDAI	3.5 (3.0, 4.0)	62.9%
**Current treatment**	csDMARDs	139 (52.1%)	0%
	bDMARDs	104 (39.0%)	0%
	tsDMARDs	20 (7.5%)	0%
	NSAIDs	94 (35.2%)	0%
	GCs	23 (8.6%)	0%
**Patient reported outcomes**	HAQ (0–3)	0.8 (0.7, 0.9)	4.9%
	MDHAQ Functional (0–3)	0.8 (0.7, 0.9)	6%
	MDHAQ Psychological (0–3)	0.9 (0.8, 0.9)	6%

**Footnote**: The results are presented as number (percentage) or mean (95% CI)

**Abbreviations:** BASDAI - Bath Ankylosing Spondylitis Disease Activity Index, bDMARDs - biologic disease-modifying antirheumatic drugs, BMI - Body mass index, CRP - C-reactive protein, csDMARDs - conventional synthetic disease-modifying antirheumatic drugs, DAPSA - Disease Activity in Psoriatic Arthritis, GCs -Glucocorticosteroids, HAQ - Health Assessment Questionnaire, MDHAQ - Multi-Dimensional Health Assessment Questionnaire, NSAIDs - Nonsteroidal anti-inflammatory drugs, tsDMARDs - targeted synthetic disease-modifying antirheumatic drugs

The mean number of comorbidities in one individual equaled 1.0 (0.8, 1.1). The most prevalent comorbidities were CVD (29.2%), endocrine/metabolic (20.2%), especially thyroid disease (13.9%) and psychiatric (10.5%)– all categories of comorbidities are presented in Table 2. Multimorbidity, defined as the presence of two or more chronic diseases in the same individual [34], was observed in 50.2% of cases of the whole group. Most of these subjects had one additional disease (except for PsA). The maximal number of comorbidities were 6 (in one case). The presence of two or more comorbidities (apart from PsA) were observed in 27.7% of cases.

**Table 2:** Detailed comorbidity characteristics of 267 psoriatic arthritis patients

		Total	Missing data (%)
**Mean number of comorbidities**		1.0 (0.8, 1.1)	0%
**Multimorbidity**		134 (50.2%)	0%
**Number of comorbidities**	**0**	133 (49.8%)	0%
	**1**	60 (22.5%)	
	**2**	45 (16.9%)	
	**3**	15 (5.6%)	
	**4**	7 (2.6%)	
	**5**	6 (2.2%)	
	**6**	1 (0.4%)	
**Cardiovascular disease**		78 (29.2%)	0%
**Endocrine/metabolic**		54 (20.2%)	0%
Diabetes *		21 (7.9%)	0%
Thyroid disease *		37 (13.9%)	0%
**Gastrointestinal disease**		22 (8.2%)	0%
**Pulmonary disease**		14 (5.2%)	0%
**Chronic infections**		9 (3.4%)	0%
**Cancer**		9 (3.4%)	0%
**Leukemia or lymphoma**		0 (0%)	0%
**Mental disease**		28 (10.5%)	0%
**Kidney disease**		9 (3.4%)	0%
**Osteoarthritis**		13 (4.9%)	0%
**Fibromyalgia**		12 (4.5%)	0%
**ENT disorders**		7 (2.6%)	0%

**Footnote**: The results are presented as number (percentage)

* Also included into ‘Endocrine/metabolic’ category

**Abbreviations**: ENT - ear, nose and throat

The scores of HAQ, MDHAQFn, and MDHAQPs in the whole group were 0.8 (0.7,0.9), 0.8 (0.7,0.9) and 0.9 (0.8,0.9), respectively – they are presented in Table 1. The results achieved in particular domains of SF-36 are displayed in Table 3. The differences in the domains of HRQoL included in SF-36 between subgroups with and without multimorbidity are presented in Table 2. HRQoL was worse in the group with multimorbidity, compared to the group without multimorbidity, with regard to bodily pain (34.7 [30.1, 39.3] vs. 47.5 [43.1, 52.0]; *p* < 0.01), physical functioning (52.1 [47.3, 56.9] vs. 63.1 [58.9, 67.4]; *p* < 0.01) and role limitations due to physical problems (28.5 [21.2, 35.9] vs. 42.8 [35.2, 50.4]; *p* < 0.01). Establishing the cutoff at two or more comorbidities (apart from PsA) in one individual, we achieved comparable results, except for general health domain, which score was significantly lower in the group with two or more comorbidities (33.4 [29.6, 37.4] vs. 39.5 [36.9, 42.1]; *p* < 0.01).

**Table 3:** Health-related quality of life explored utilizing SF-36, described in participants, also dichotomizing focusing on the presence of multimorbidity

		Multimorbidity 134 patients (50.2%)	No multimorbidity 133 patients (40.8%)	Missing data (%)	*p*-value
**SF-36**	**Mental health**	59.3 (55.9, 62.7)	59.7 (56.5, 62.8)	14.6%	0.78
	**Vitality**	44.9 (41.3, 48.4)	49.4 (45.9, 52.8)	14.6%	0.17
	**Bodily pain**	34.7 (30.1, 39.3)	47.5 (43.1, 52.0)	14.6%	**< 0.01**
	**General health**	35.9 (32.8, 38.9)	39.8 (36.8, 42.9)	14.6%	0.09
	**Social functioning**	57.9 (53.4, 62.3)	62.6 (58.2, 67.0)	14.6%	0.21
	**Physical functioning**	52.1 (47.3, 56.9)	63.1 (58.9, 67.4)	14.6%	**< 0.01**
	**Role limitations due to physical problems**	28.5 (21.2, 35.9)	42.8 (35.2, 50.4)	14.6%	**< 0.01**
	**Role limitations due to emotional problems**	51.6 (43.2, 60.1)	53.6 (45.7, 61.6)	14.6%	0.68
		Presence of 2 or more comorbidities* **74 patients (27.7%)**	Presence of 0 or 1 comorbidities * **193 patients (72.3%)**	**Missing data (%)**	***p-value***
**SF-36**	**Mental health**	60.0 (55.4, 64.6)	58.9 (56.2, 61.6)	14.6%	0.49
	**Vitality**	43.1 (38.0, 48.2)	48.6 (45.8, 51.4)	14.6%	0.11
	**Bodily pain**	30.5 (24.8, 36.2)	45.1 (41.3, 48.9)	14.6%	**< 0.01**
	**General health**	33.4 (29.6, 37.4)	39.5 (36.9, 42.1)	14.6%	**< 0.01**
	**Social functioning**	56.6 (50.3, 62.8)	61.6 (58.0, 65.2)	14.6%	0.27
	**Physical functioning**	49.6 (43.5, 55.6)	60.6 (56.8, 64.4)	14.6%	**< 0.01**
	**Role limitations due to physical problems**	21.7 (12.9, 30.5)	40.9 (34.5, 47.3)	14.6%	**< 0.01**
	**Role limitations due to emotional problems**	56.8 (45.3, 68.3)	51.1 (44.4, 57.8)	14.6%	0.43

**Footnote**: The results are presented as numbers (percentages) or means (95% CI). The Mann-Whitney U test was used to identify statistically significant differences between groups

Multimorbidity is defined as living with two or more chronic diseases, including psoriatic arthritis

*Apart from psoriatic arthritis

**Abbreviations:** SF-36–36-Item Short Form Survey

The effect of distinct parameters, including demographic parameters, comorbidities and number of comorbidities on HAQ, MDHAQFn, MDHAQPs and particular domains of SF-36 is shown in Tables 4 and 5. Female sex was inversely correlated with MDHAQPs score (β= -0.24, *p* < 0.01) and positively with MH score (β = 0.15; *p* = 0.04), as well as RE score (β = 0.17, *p* = 0.04). Higher BMI score was associated with higher HAQ score (β = 0.12, *p* = 0.04) and lower results of BP and PF categories. The presence of CVD was positively correlated with MDHAQFn score (β = 0.17, *p* < 0.01) and inversely with RE score (β= -0.27, *p* = 0.02). Lower RE score was also associated with diabetes (β= -0.24, *p* < 0.01). Mental health disorders negatively influenced MH (β= -0.35, *p* < 0.01), VT (β= -0.22, *p* < 0.01), GH (β= -0.19, *p* < 0.01), SF (β= -0.15, *p* = 0.04) and RE (β= -0.30, *p* < 0.01). Number of comorbidities was inversely correlated with MH (β = 0.34, *p* < 0.01) and RE (β = 0.59, *p* < 0.01). Higher DAPSA score was associated with all of the analysed PROs (HAQ, MDHAQFn, MDHAQPs, SF-36 domains).

**Table 4:** Factors Influencing Patient-Reported Outcome Measures: results of adjusted analysis

Variables included in primary model	HAQ		MDHAQ Functional		MDHAQ Psychological	
	β	*p*-value	β	*p*-value	β	*p*-value
**Female sex**	-0.11	0.06			-0.24	**< 0.01**
**Age**			-0.10	0.09		
**BMI**	0.12	**0.04**				
**Disease duration**						
**DAPSA**	0.59	**< 0.01**	0.63	**< 0.01**	0.36	**< 0.01**
**Cardiovascular disease**	0.11	0.09	0.17	**< 0.01**		
**Diabetes**						
**Thyroid disease**					-0.10	0.17
**Gastrointestinal disease**	-0.11	0.06	-0.12	**0.04**	-0.12	0.08
**Pulmonary disease**			0.09	0.12		
**Chronic infections**						
**Cancer**						
**Leukemia or lymphoma**						
**Mental disease**						
**Kidney disease**					0.09	0.19
**Osteoarthritis**			-0.10	0.11		
**Fibromyalgia**						
**ENT disorders**	0.10	0.09				
**Number of comorbidities**						
**Adjusted R2**	0.45		0.44		0.21	

**Footnote**: The coefficients are derived from multivariable linear regression with backward elimination, using an F-to-remove value greater than 0.20 for variable removal. The adjusted R² was then calculated based on the remaining variables in the models. Gray fields indicate the variables removed from the final model due to their low significance

**Abbreviations:***β*- adjusted beta coefficient, BMI - Body mass index, DAPSA - Disease Activity in Psoriatic Arthritis, ENT - ear, nose and throat, HAQ - Health Assessment Questionnaire, MDHAQ - Multi-Dimensional Health Assessment Questionnaire

**Table 5:** Factors associated with components of the SF-36 instrument - adjusted analysis

Variables included in primary model*	SF-36 domains															
	MH		VT		BP		GH		SF		PF		RP		RE	
	β	*p*-value	β	*p*-value	β	*p*-value	β	*p*-value	β	*p*-value	β	*p*-value	β	*p*-value	β	*p*-value
**Female sex**	0.15	**0.04**													0.17	**0.04**
**BMI**	0.10	0.17			-0.18	**< 0.01**			0.11	0.12	-0.21	**< 0.01**				
**Disease duration**					0.17	**0.01**	0.09	0.20								
**DAPSA**	-0.25	**< 0.01**	-0.29	**< 0.01**	-0.45	**< 0.01**	-0.29	**< 0.01**	-0.23	**< 0.01**	-0.51	**< 0.01**	-0.49	**< 0.01**	-0.22	**< 0.01**
**Cardiovascular disease**															-0.27	**0.02**
**Diabetes**	-0.10	0.20			-0.10	0.12							-0.12	0.06	-0.24	**< 0.01**
**Mental disease**	-0.35	**< 0.01**	-0.22	**< 0.01**			-0.19	**< 0.01**	-0.15	**0.04**					-0.30	**< 0.01**
**Number** **of comorbidities**	0.34	**< 0.01**													0.59	**< 0.01**
**Adjusted R2**	0.16		0.16		0.27		0.14		0.10		0.31		0.27		0.11	

**Footnote**: The coefficients are derived from multivariable linear regression with backward elimination, using an F-to-remove value greater than 0.20 for variable removal. The adjusted R² was then calculated based on the remaining variables in the models. Gray fields indicate the variables removed from the final model due to their low significance

*Following variables were included in the model but are not displayed, because their associations did not reach statistical significance: Age, Thyroid disease, Gastrointestinal disease, Pulmonary disease, Chronic infections, Cancer, Leukemia or lymphoma, Kidney disease, Osteoarthritis, Fibromyalgia, ENT disorders

**Abbreviations:***β*- adjusted beta coefficient, BMI - body mass index, BP - bodily pain, DAPSA - Disease Activity in Psoriatic Arthritis, ENT - ear, nose and throat, GH – general health, MH – mental health, PF- physical functioning, RE – role limitations due to emotional problems, RP – role limitations due to physical problems, SF – social functioning, SF – 36–36-Item Short Form Health Survey, VT – vitality

## Discussion

Based on our data, we assessed the prevalence of comorbidities, which seems to be comparable to literature data [11]. Mean number of comorbidities in one individual equaled one and multimorbidity was present in 50.2% cases. Varied results were observed in the other reports [35–38]. We found that multimorbidity was associated with poorer QoL, measured by SF-36 in the domains associated with physical health, pain and role limitations due to physical and emotional problems. The study by Radner et al. showed similar results on the impact of comorbidities on SF-36 in rheumatoid arthritis, revealing their influence on physical but not mental functioning [39]. Poorer outcomes of both physical and mental dimensions in SF-36 scores in PsA groups with multimorbidity were evaluated in the study by Husted et al. However, a cutoff point for multimorbidity group was set at 3 or more comorbidities [35].

CVD are known to be associated with morbidity and mortality in PsA [40]. In the regression model we found the association of CVD with poorer results of MDHAQFn. Decreased level of physical health in association with CVD was reported in another study [35]. Obesity was one of the comorbidities most strongly associated with decreased physical health in the aforementioned paper [35]. In the study by Zaffarana et al., obesity was associated with higher levels of pain and worse functional capacity [41]. We found that the group with a higher BMI score achieved higher HAQ results, as well as lower scores in BP and PF domains of SF-36. In another study, patients with PsA and more than one comorbidity have poorer physical function, measured by HAQ score [42]. Our analysis also indicated the poorer results of MH, RE, SF, GH and VT in SF-36 domains in cases with psychiatric disorders. Similarly, a reduction in mental function was reported in the study by Husted et al. [35]. The correlation of depression and anxiety with physical aspects of QoL was present in another report [43]. However, our regression model, assessing the impact of mental health on MDHAQFn and MDHAQPs did not reveal correlation.

We did not find the association of the number of comorbidities with functional outcomes (HAQ, MDHAQFn) or HRQoL (SF-36 domains). These results are similar to the other reports which stated that the type of comorbidity had greater impact on QoL than the number of comorbidities [35, 38]. On the contrary, in a systematic review assessing the impact of comorbidities on PROs, patients with a higher number of comorbidities reported a worse impact of their disease on, among the others, pain, fatigue, function, work disability, and QoL [29]. Unexpectedly, the correlation of number of comorbidities with psychological dimensions of SF-36 (MH, RE) was inverse.

We did not show the influence of female sex on physical functioning in our analysis, contrary to other reports indicating negative impact [42, 44]. Moreover, female patients had better results of MDHAQPs and mental health domains of SF-36 (MH, RE) in our group. The association of DAPSA score with the results of PROs suggests the significant impact of disease activity on patient-reported health status and QoL [45].

Demographic data of our PsA group are generally similar to data from the other reported populations [41, 46–49]. Clinical indices, as well as PROs’ scores, indicate a low to moderate burden of disease. The three most prevalent groups of comorbidities were CVD, endocrine/metabolic (including diabetes), and psychiatric disorders. In a large meta-analysis [11], the most frequently seen comorbidities in PsA were hypertension and other CVD as well as metabolic disorders, including obesity, hyperlipidemia, and diabetes mellitus. The findings in a recently published narrative review indicated CVD, metabolic, and mental health disorders as the most prevalent comorbidities [13]. Generally, the kind and frequency of reported comorbidities in our population are similar to the other literature data.

To our knowledge, this is the first study assessing the impact of comorbidities on PROs in PsA in the Polish population. The strength of our study is the number of cases included, which makes performed analyses reliable. The data were collected using validated tools and during routine visits, constituting high-quality, real-world data. As there is a lack of studies evaluating the impact of comorbidities on PROs in PsA as the main goal of the study [29], our data might additionally contribute to the increase of knowledge on this matter. On the other hand, the study has several limitations. Firstly, it was performed in a cross-sectional manner. Therefore, we can compare the results of PROs in the presence or absence of the comorbidities at one point in time, but we cannot assess the prospective impact of comorbidities on the change of PROs results. However, the development of a prospective database for collecting future data may allow further analyses to be carried out. Secondly, there are some missing data regarding clinical parameters and PROs in the analysed population, which may result in bias. Nevertheless, the proportion of missing data is small, reaching only 6%, with regard to crucial questionnaires. Additionally, there are some differences in the classification of distinct comorbidities, which may render the comparisons with the other reports more difficult. Moreover, the coefficients of determination (R^2^) in our regression models are rather low, which suggests poor prediction value of the model. Finally, we evaluated the impact of wider subgroups of comorbidities, the presence of multimorbidity or number of comorbidities on PROs, however the severity and burden of particular diseases from one category might be different.

In conclusion, our data are consistent with the other reports with regard to the type and prevalence of comorbidities in PsA. Multimorbidity is present in about a half of cases. There is a significant impact of multimorbidity on physical aspects of QoL, including physical activity, role limitation due to physical disability and feeling of pain. CVD adversely influence functional capacity measured by MDHAQFn, while mental health disorders are associated with poorer outcomes, regarding general health, mental health and social functioning. In addition, higher BMI is associated with poorer physical functioning and greater feeling of pain. The impact of comorbidities should be taken into account by clinicians and researchers assessing PROs.

### Electronic supplementary material

Below is the link to the electronic supplementary material.

Supplementary Material 1

## Data Availability

The data presented in the study are available on a reasonable request from the corresponding author.

## References

[1] Coates LC, Helliwell PS (2017). Psoriatic arthritis: state of the art review. Clin Med (Northfield Il).

[2] Lipton S, Deodhar A (2012). The new ASAS classification criteria for axial and peripheral spondyloarthritis: promises and pitfalls. Int J Clin Rheumatol.

[3] Scotti L, Franchi M, Marchesoni A, Corrao G (2018). Prevalence and incidence of psoriatic arthritis: a systematic review and meta-analysis. Semin Arthritis Rheum.

[4] Lembke S, Macfarlane GJ, Jones GT (2024) The worldwide prevalence of psoriatic arthritis-a systematic review and meta-analysis. Rheumatology (Oxford). 26:keae198 Epub ahead of print 10.1093/rheumatology/keae198 PMC11637478 38530786

[5] Gladman DD, Shuckett R, Russell ML, Thorne JC, Schachter RK (1987). Psoriatic arthritis (PSA)--an analysis of 220 patients. Q J Med.

[6] Ritchlin CT, Colbert RA, Gladman DD (2017). Psoriatic arthritis. N Engl J Med.

[7] Elalouf O, Muntyanu A, Polachek A, Pereira D, Ye JY, Lee KA (2020). Mortality in psoriatic arthritis: risk, causes of death, predictors for death. Semin Arthritis Rheum.

[8] Ramiro S, Nikiphorou E, Sepriano A, Ortolan A, Webers C, Baraliakos X (2022). ASAS-EULAR recommendations for the management of axial spondyloarthritis: 2022 update. Ann Rheum Dis.

[9] Gossec L, Baraliakos X, Kerschbaumer A, De Wit M, McInnes I, Dougados M (2020). EULAR recommendations for the management of psoriatic arthritis with pharmacological therapies: 2019 update. Ann Rheum Dis.

[10] FitzGerald O, Ogdie A, Chandran V, Coates LC, Kavanaugh A, Tillett W (2021). Psoriatic arthritis. Nat Rev Dis Primers.

[11] Gupta S, Syrimi Z, Hughes DM, Zhao SS (2021). Comorbidities in psoriatic arthritis: a systematic review and meta-analysis. Rheumatol Int.

[12] Kaine J, Song X, Kim G (2019). Higher incidence rates of comorbidities in patients. J Manag Care Spec Pharm.

[13] Panagiotopoulos A, Fragoulis GE (2023) Comorbidities in Psoriatic Arthritis: A Narrative Review. ; 45 (2):177 – 89 10.1016/j.clinthera.2023.01.006 36737317

[14] Husni ME (2015). Comorbidities in psoriatic arthritis. Rheum Dis Clin North Am.

[15] Zhao SS, Miller N, Harrison N, Duffield SJ, Dey M, Goodson NJ (2020). Systematic review of mental health comorbidities in psoriatic arthritis. Clin Rheumatol.

[16] Lubrano E, Scriffignano S, Perrotta FM (2020). Multimorbidity and comorbidity in psoriatic arthritis - a perspective. Expert Rev Clin Immunol.

[17] Stober C, Ye W, Guruparan T, Htut E, Clunie G, Jadon D (2018). Prevalence and predictors of tumour necrosis factor inhibitor persistence in psoriatic arthritis. Rheumatology (Oxford).

[18] Her M, Kavanaugh A (2012). Patient-reported outcomes in rheumatoid arthritis. Curr Opin Rheumatol.

[19] Gossec L, Dougados M, Dixon W (2015). Patient-reported outcomes as end points in clinical trials in rheumatoid arthritis. RMD Open.

[20] Chakravarty SD, Abell J, Leone-Perkins M, Orbai AM (2021). A Novel qualitative study assessing patient-reported outcome measures among people living with Psoriatic Arthritis or Ankylosing Spondylitis. Rheumatol Ther.

[21] Kalyoncu U, Dougados M, Daurès JP, Gossec L (2009). Reporting of patient-reported outcomes in recent trials in rheumatoid arthritis: a systematic literature review. Ann Rheum Dis.

[22] Bruce B, Fries JF (2003). The Stanford Health Assessment Questionnaire: dimensions and practical applications. Health Qual Life Outcomes.

[23] Blackmore MG, Gladman DD, Husted J, Long JA, Farewell VT (1995). Measuring health status in psoriatic arthritis: the Health Assessment Questionnaire and its modification. J Rheumatol.

[24] Mease P, Strand V, Gladman D (2018). Functional impairment measurement in psoriatic arthritis: importance and challenges. Semin Arthritis Rheum.

[25] Pincus T, Swearingen C, Wolfe F (1999). Toward a multidimensional Health Assessment Questionnaire (MDHAQ): Assessment of advanced activities of daily living and psychological status in the patient-friendly health assessment questionnaire format. Arthritis Rheum.

[26] Ye W, Hackett S, Vandevelde C, Twigg S, Helliwell PS, Coates LC (2021). Measuring physical function in psoriatic arthritis: comparing the Multidimensional Health Assessment Questionnaire to the Health Assessment Questionnaire-Disability Index. J Rheumatol.

[27] Husted JA, Gladman DD, Farewell VT, Cook RJ (2001). Health-related quality of life of patients with psoriatic arthritis: a comparison with patients with rheumatoid arthritis. Arthritis Care Res.

[28] Leung YY, Tillett W, Hojgaard P, Orbai AM, Holland R, Mathew AJ (2021). Test-retest reliability for HAQ-DI and SF-36 PF for the measurement of physical function in psoriatic arthritis. J Rheumatol.

[29] Cañete JD, Tasende JAP, Laserna FJR, Castro SG, Queiro R (2020). The impact of Comorbidity on patient-reported outcomes in psoriatic arthritis: a systematic literature review. Rheumatol Ther.

[30] Taylor W, Gladman D, Helliwell P, Marchesoni A, Mease P, Mielants H, CASPAR Study Group (2006). Classification criteria for psoriatic arthritis: development of new criteria from a large international study. Arthritis Rheum.

[31] Haugeberg G, Hansen IJ, Soldal DM, Sokka T (2015). Ten years of change in clinical disease status and treatment in rheumatoid arthritis: results based on standardized monitoring of patients in an ordinary outpatient clinic in southern Norway. Arthritis Res Ther.

[32] Steyerberg E (2010) Clinical Prediction Models: A Practical Approach to Development, Validation and Updating by STEYERBERG, E. W. Vol. 66, Biometrics. 661–662 p

[33] Hair J, Babin RA, Black B (2014) W. Multivariate Data Analysis.pdf. Vol. 7 edition, Australia: Cengage. p. 758

[34] Radner H, Yoshida K, Smolen JS, Solomon DH (2014). Multimorbidity and rheumatic conditions - enhancing the concept of comorbidity. Nat Rev Rheumatol.

[35] Husted JA, Thavaneswaran A, Chandran V, Gladman DD (2013). Incremental effects of comorbidity on quality of life in patients with psoriatic arthritis. J Rheumatol.

[36] Nas K, Karkucak M, Durmus B, Karatay S, Capkin E, Kaya A (2015). Comorbidities in patients with psoriatic arthritis: a comparison with rheumatoid arthritis and psoriasis. Int J Rheum Dis.

[37] Chanakya K, Shobha V, Chandrashekara S, Kumar S, Haridas V, Rao V (2023). Comorbidity burden in psoriatic arthritis and its impact on disease measures. Indian J Rheumatol.

[38] Bavière W, Deprez X, Houvenagel E, Philippe P, Deken V, Flipo RM (2020). Association between comorbidities and quality of life in psoriatic arthritis: results from a multicentric cross-sectional study. J Rheumatol.

[39] Radner H, Smolen JS, Aletaha D (2011). Comorbidity affects all domains of physical function and quality of life in patients with rheumatoid arthritis. Rheumatology.

[40] Jamnitski A, Symmons D, Peters MJL, Sattar N, Mcilnnes I, Nurmohamed MT (2013). Cardiovascular comorbidities in patients with psoriatic arthritis: a systematic review. Ann Rheum Dis.

[41] Zaffarana C, Yanzi JG, Cerda OL, Landi M, Schneeberger E, Citera G (2016). Prevalence of obesity in patients with psoriatic arthritis and its impact on the severity of the disease. Arthritis Rheumatol.

[42] Fernández-Carballido C, Martín-Martínez MA, García-Gómez C, Castañeda S, González-Juanatey C, Sánchez-Alonso F (2020). Impact of comorbidity on physical function in patients with ankylosing spondylitis and psoriatic arthritis attending rheumatology clinics: results from a cross-sectional study. Arthritis Care Res.

[43] Kotsis K, Voulgari PV, Tsifetaki N, Machado MO, Carvalho AF, Creed F (2012). Anxiety and depressive symptoms and illness perceptions in psoriatic arthritis and associations with physical health-related quality of life. Arthritis Care Res (Hoboken).

[44] Eder L, Thavaneswaran A, Chandran V, Gladman DD (2013). Gender difference in disease expression, radiographic damage and disability among patients with psoriatic arthritis. Ann Rheum Dis.

[45] Lo Monaco M, Mallaci Bocchio R, Natoli G, Giardina A, Cangemi I, Scibetta S (2023). Critical importance of patient-reported outcomes for a comprehensive assessment of psoriatic arthritis patients. Front Med.

[46] Tanner S, McFadden M, Clegg D, Walsh J (2014). Relationship between psoriatic arthritis severity, duration, and comorbidities. Arthritis Rheumatol.

[47] Ballegaard C, Hojgaard P, Dreyer L, Cordtz R, Jørgensen TS, Skougaard M (2018). Impact of comorbidities on tumor necrosis factor inhibitor therapy in psoriatic arthritis: a population-based cohort study. Arthritis Care Res (Hoboken).

[48] Bremander A, Jacobsson LT, Bergman S, Haglund E, Lofvendahl S, Petersson IF (2015). Smoking is associated with a worse self-reported health status in patients with psoriatic arthritis: data from a Swedish population-based cohort. Clin Rheumatol.

[49] Piche M, Chandran V, Gladman D, Rohekar S (2015). The effect of cigarette smoking and alcohol intake on patient reported outcome measures in psoriatic arthritis. J Rheumatol.

